# Socioeconomic Inequalities in Green Space Quality and Accessibility—Evidence from a Southern European City

**DOI:** 10.3390/ijerph14080916

**Published:** 2017-08-15

**Authors:** Elaine Hoffimann, Henrique Barros, Ana Isabel Ribeiro

**Affiliations:** 1 EPIUnit–Instituto de Saúde Pública, Universidade do Porto, 4050-600 Porto, Portugal; elainehoffimann@gmail.com; 2 Departamento de Ciências da Saúde Pública e Forenses e Educação Médica, Faculdade de Medicina, Universidade do Porto, 4200-319 Porto, Portugal; hbarros@med.up.pt

**Keywords:** urban health, green areas, built environment, physical activity, environmental justice

## Abstract

***Background***: The provision of green spaces is an important health promotion strategy to encourage physical activity and to improve population health. Green space provision has to be based on the principle of equity. This study investigated the presence of socioeconomic inequalities in geographic accessibility and quality of green spaces across Porto neighbourhoods (Portugal). ***Methods***: Accessibility was evaluated using a Geographic Information System and all the green spaces were audited using the Public Open Space Tool. Kendall’s tau-b correlation coefficients and ordinal regression were used to test whether socioeconomic differences in green space quality and accessibility were statistically significant. ***Results***: Although the majority of the neighbourhoods had an accessible green space, mean distance to green space increased with neighbourhood deprivation. Additionally, green spaces in the more deprived neighbourhoods presented significantly more safety concerns, signs of damage, lack of equipment to engage in active leisure activities, and had significantly less amenities such as seating, toilets, cafés, etc. ***Conclusions***: Residents from low socioeconomic positions seem to suffer from a double jeopardy; they lack both individual and community resources. Our results have important planning implications and might contribute to understanding why deprived communities have lower physical activity levels and poorer health.

## 1. Introduction

Physical activity (PA) is an important health determinant, being associated with numerous health benefits [1,2,3]. Physical activity habits are influenced by a myriad of aspects that operate at both the individual and environmental level [4]. Among the environmental correlates of PA, green space seems to play a prominent role. According to the last Eurobarometer survey on Sport and Physical Activity, green spaces are the preferable location to engage in PA: of the 20,912 adult respondents, 40% reported to engage in PA in parks and outdoors, whereas a smaller percentage reported to use health or fitness centres (15%) [5]. Findings from epidemiological research confirm these reports: people that reside near green spaces spend more time in leisure PA [6,7] and are more likely to achieve the recommended amount of daily PA [8].

Therefore, the provision of green spaces is an important health promotion strategy to encourage PA [9,10,11] and to improve health in general, as the benefits of green space go far beyond those related to PA [12]. Green space provision, however, has to be based on the principle of equity, ensuring public access for the entire population regardless of an individual’s residential location, socioeconomic background, or ethnicity/race [13,14,15]. This is especially relevant since there is a stark socioeconomic gradient in PA [16] and in other health concerns [17]. Socioeconomic inequalities in PA have also been documented according to neighbourhood deprivation, even after adjusting for individual-level socioeconomic position [18,19]. Such differentials may be in part related to the fact that deprived communities lack access to physical activity resources, such as green spaces (the “deprivation-amplification” hypothesis) [20].

Therefore, it is critical to assess and monitor whether all population strata, regardless of their socioeconomic background, have access to green space. But it is important to bear in mind that the mere presence of a green space does not guarantee its use; access to green space depends not only on the geographic proximity or accessibility (i.e., the presence of green space within a reasonable distance from home), but also on green space quality (i.e., presence and quality of facilities and amenities) [21,22,23]. These two dimensions must be considered when measuring socioeconomic inequalities in green space provision and should preferably be evaluated using objective assessments and methods [22].

A considerable number of studies have evaluated potential disparities in green space accessibility and quality. Yet, findings vary considerably from context to context [9,22], indicating that it is not possible to extrapolate results to other parts of the world or even other cities in the same country. Some studies reported that socioeconomically disadvantaged and minority neighbourhoods are equipped with fewer green spaces and present more quality and safety concerns [24,25,26,27,28,29,30,31,32]. Others found that although geographic accessibility to green space does not differ according to neighbourhood deprivation, green space size and quality are considerably worse in the most deprived neighbourhoods [33,34,35,36]. Nevertheless, a fair amount of investigations concluded that both accessibility and quality of green space are fairly distributed, regardless of the socioeconomic background of the communities [22,37,38,39,40,41]. Moreover, some investigations even showed that green space distribution favoured minorities and low-income groups [22,42]. Not only is the evidence mixed, but also it is strongly geographically biased. Previously mentioned studies come almost exclusively from Anglo-Saxon countries: USA, Australia, New Zealand, and the UK. This is likely a consequence of the geographic origins of the environmental justice movement, which was born in the USA in the mid-1980s [43,44], and has been mostly disregarded in Europe (apart from the UK), generally assumed to be a less unequal territory [45,46]. However, recent research has found large between-city differences in green space availability across European settings [47], where southern European cities showed below-average availability values.

In the Portuguese context, several studies have found large socioeconomic inequalities in health in Portuguese settings [48], and specifically in Porto municipality [18,49], which despite being relatively small in territorial extent, holds important socioeconomic disparities and presents a high degree of socio-spatial segregation, as most of the population and neighbourhoods are situated either in the least or in the most deprived areas [49,50]. Also, the urbanization process that accelerated after the 20th century in the city caused fragmentation and discontinuity in the green infrastructure, meaning that in only a century, Porto transformed from a “green city” (76% of the city was covered by green area in 1892) to a “grey city” (29% in 2000) [51]. Alongside this change, physical activity levels of the Portuguese and Porto residents remain amongst the lowest in Europe [5]. For instance, among the older adults, only 48% engage in some form of leisure activity, and only 3% of the women and 33% of the men achieve the recommended weekly amount of exercise [6]. Thus, it is important to assess whether green space is equally distributed across city and, so far, no study addressed this issue.

In brief, there is a lack of research that address the presence of socioeconomic differentials in green space provision, evaluated using both accessibility and quality-based measures, in Europe and specifically in Portugal. Thus, aiming at assisting planning interventions at local level, the purpose of the present study was to assess whether there are socioeconomic inequalities in geographic accessibility and quality of green spaces across Porto neighbourhoods using objective and recently collected data reporting to the year of 2016.

## 2. Materials and Methods

### 2.1. Study Area

The Porto municipality is located in the northwest of Continental Portugal and is comprised of approximately 215,000 inhabitants [52], distributed across 41.7 km^2^. Porto is limited by the Atlantic coast, and extends along the Douro River estuary. It is an industrial and port town within the Porto Metropolitan Area, the second largest metropolitan area of Portugal, with roughly 1.3 million inhabitants.

The Porto municipality is divided into 2064 census tracts, each of them holding a median of 95 inhabitants. Census tracts are an operational unit for Census data collection (the reason why they hold a very balanced number of inhabitants) and constitute the smallest geographical unit of census data dissemination [53]. Census tracts from here onwards simply referred to as neighbourhoods, were used as unit of analysis in the present study.

### 2.2. Green Spaces

**Figure 1:**
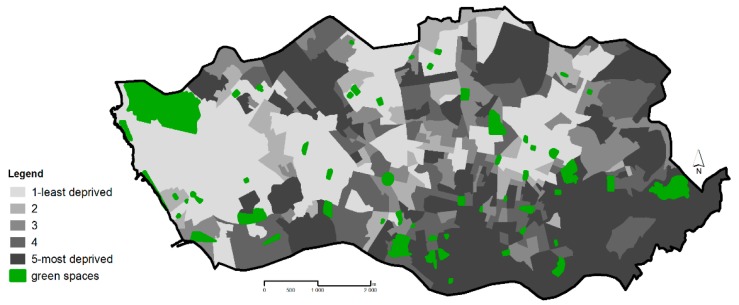
Geographic distribution of the public green spaces and neighbourhood socioeconomic deprivation in Porto municipality.

The city of Porto has 55 public green spaces, 52 of which are administered by the Porto city council, and three are privately owned but freely accessible to the public, from which seven correspond to urban parks, 12 to historical gardens and 36 to proximity green spaces. Our sample was constituted of these 55 green spaces, without restrictions of size, location or characteristics, to cover the universe of public green spaces available in the city that can be freely used by the population to engage in leisure and physical activities. Green space polygons and entrance locations were obtained from the city council digital maps as in previous studies by the team [6,7]. The geographic distribution of Porto green spaces is depicted in Figure 1.

### 2.3. Geographic Accessibility to Green Spaces

There is no consensus on the maximum pedestrian distance people are willing to walk to use a green space; distances used in the literature range from 1 mile to 1/4 mile [33,54]. In this study, we used the 800 m (1/2 mile, equivalent to a 10 min walk) threshold as employed in other studies focused on measuring pedestrian access to neighbourhood facilities, namely green spaces [24,55,56,57].

Using the centroid of each neighbourhood as the unit of analysis, we computed the following measures of geographic accessibility to green spaces: (1) availability (or not) of green spaces within an 800 m road distance; (2) number of green spaces within an 800 m road distance; (3) mean distance (in hectometre, hm) to the green spaces within 800 m; and (4) area (squared meters) of green space per inhabitant within an 800 m road distance.

To estimate the above mentioned measures of geographic accessibility we used the ArcGIS 10.4 Network Analyst tool (Environmental Systems Research Institute, ESRI, Redlands, CA, USA) and an updated street network dataset provided courtesy of ESRI (Redlands, CA, USA). The geometric centroid of each neighbourhood was used as starting point to identify the green spaces that were within the established 800 m threshold distance. Whenever green spaces were bounded we used the distance to the entrance; otherwise the distance to the boundary was considered.

To assess the robustness of our findings and evaluate in what extent the chosen threshold distance was driving our results, a sensitivity analysis were conducted, where the distance cut-off of 400 m (1/4 mile, also often employed) was considered; despite the 40% reduction in the number of neighbourhoods with accessible green space, results remained mostly unchanged (Table S2).

### 2.4. Green Space Quality

The 55 green spaces from Porto were audited using the Public Open Space Tool (POST) [58]. The POST is a fast, well-established, and validated tool designed to audit public open spaces, such as parks and other green spaces, with a special emphasis on attributes that may encourage or discourage its use for physical activity [59]. It is based on direct observation and consists of 49 elements covering four key domains: activities (e.g., type of use and specific activities for which the space was designed), environmental quality (e.g., presence of attractive elements, such as trees, statues, water), comfort (e.g., presence of amenities, such as cafés, parking lots, public restrooms), and safety (e.g., presence of illumination, signs and characteristics of the surrounding roads) [60].

Audits in the field were carried out from August to November 2016 by a trained researcher. Immediately after, a second audit was carried out by another researcher to evaluate inter-rater reliability. Moderate to strong agreement levels were observed (Cohen’s kappa between 0.67 and 0.96) [61].

In the present study, we used 32 out of the 49 items from POST. We excluded items that were absent (e.g., barbecue area, dog fountains) and/or present (e.g., presence trees, access to public transport) in all green spaces, as they did not discriminate green spaces, and items with an unpredictable impact in green space use (e.g., arrangement of paths and trees). Additionally, to support these decisions of the present investigation, an extensive literature review about the green space attributes that are preferred by the population was conducted.

Besides looking at each individual POST item, we computed a summary measure of green space quality, which involved variable recodification and the calculation of an overall quality score and quality scores by domain (activities, environmental quality, comfort and safety). First, multi-category and continuous variables were recoded into dichotomous variables by aggregating categories or dividing the variable based on the median, respectively. For example, the question “is there a kiosk/café present?” which allowed four possible answers (7 days per week/Weekdays only/Weekends only/No) was transformed into a dichotomous variable, where 0 corresponded to “no kiosk or café” and 1 to “yes there is a kiosk or café”, regardless the schedule. Additionally, variables about detrimental features (e.g., presence of vandalism) were recoded so that 1 corresponded to the absence of that feature and 0 corresponded to the presence of that feature. As usual, variables on beneficial features were coded 1 (feature is present) and 0 (feature is absent). The list of included and excluded variables, as well as recodification procedures, is provided in supplementary material (Table S1). Similar procedures were applied in previous investigations [62].

The green space quality scores resulted from the sum of the dichotomous answers (0 and 1), so that the higher the score, the higher the quality of the greenspace. The overall greenspace quality score resulted from the sum of the 32 POST items, and the scores by domain resulted from the sum of the items pertaining to each of the POST domains. Also, to take into account that the domains of POST were constituted by an unequal number of items, another version of the overall green space quality score was constructed, as follows: (i) the items pertaining to each domain were summed to obtain a score for each of the four domains; (ii) for each domain, the previously-obtained scores were ranked into terciles, where a value of 3 was assigned to greenspaces in the upper tercile, a value of 2 was assigned to those in the middle tercile, and a value of 1 was assigned to those in the lower tercile; (iii) the four ranks were summed to obtain an overall a score that could range from 4 to 12.

### 2.5. Neighbourhood Socioeconomic Deprivation

The European Deprivation Index (EDI) was used to classify the neighbourhoods according to their level of socioeconomic deprivation. The EDI is a transnational multivariate index developed for five European countries: France, England, Italy, Spain, and Portugal [63].

The EDI was constructed in three steps using both individual and area level census data. These steps are detailed elsewhere [48] but in brief were: (1) construction of an individual level indicator of deprivation, based on EU-Statistics on Income and Living Conditions (EU-SILC) information; (2) identification of variables available both at the individual level (EU-SILC) and at an area level (2001 national population census); and (3) determination, at individual level, whether the set of area-level variables from the census selected at Step 2 were associated with the indicators of individual deprivation created in Step 1. The associated census variables were then included in the formula for EDI. The final EDI score was based upon the weighted sum of these variables. The weights were the regression coefficients that quantified the association between the indicator of individual deprivation and the variables from the census that were also available at individual level identified at Step 2. The index was then categorized into quintiles (Quintile 1, Q1, to Quintile 5, Q5). The geographic distribution of the socioeconomic deprivation quintiles is depicted in Figure 1. Because the EDI and its quintiles were computed at national level, there was an unbalanced number of neighbourhoods in each of the socioeconomic deprivation quintiles in Porto as result of the high degree of socio-spatial segregation in the city.

### 2.6. Statistical Analysis

To compare greenspace accessibility and quality according to neighbourhood socioeconomic deprivation quintiles we first computed descriptive statistics: medians and IQR (interquartile range) or means and standard deviations (SD) for continuous variables, and proportions for categorical variables.

Then, to test whether greenspace accessibility and quality were significantly different according to neighbourhood socioeconomic deprivation quintiles, we used both Kendall’s tau-b correlation coefficient and ordinal regression. Kendall’s tau-b is a non-parametric rank test of correlation based on the number of concordances and discordances in paired observations [64,65]. Kendall’s tau-b correlation coefficient can be applied to measure the strength and direction of association between either categorical-ordinal variable (such as the neighbourhood deprivation quintiles) and/or non-normally distributed continuous variables (such as presence of a certain attribute). Kendall’s tau-b correlation coefficients are generally lower than Spearman’s rho correlation coefficients. Kendall's tau-b correlation coefficient was used to measure the association between individual items from POST and neighbourhood socioeconomic deprivation quintiles.

Univariable and multivariable ordinal regression were used to measure the association between neighbourhood deprivation quintiles (ordinal outcome) and the measures of green space accessibility and the domain-specific quality scores. Associations were expressed as odds ratio (OR) and 95% confidence intervals (95% CI).

Statistical significance was predetermined to be met achieved at a significance level of 0.05. All statistical analyses were conducted using SPSS 22.0 (IBM Corp., Armonk, NY, USA). Specifically, the SPSS extension Polytomous Universal Model (PLUM) was used to fit the ordinal regression models.

## 3. Results

Table 1 shows the variables related to geographic accessibility to green spaces according to quintiles of neighbourhood socioeconomic deprivation. We observed that most of the neighbourhoods, 80.2%, had a green space within a distance of 800 m, but this proportion was significantly different across quintiles of neighbourhood deprivation (OR = 0.550, 95% CI: 0.451, 0.672), with larger proportions in the least deprived neighbourhoods (Q1 = 90.0%) and smaller proportions in the most deprived (Q5 = 75.8%). Similar gradients were observed for other measures of geographic accessibility: compared with the most deprived neighbourhoods, the number of accessible green spaces was largest in the least deprived (Q1 = 2.22 versus Q5 = 1.90; OR = 0.924, 95% CI: 0.885, 0.965), and the distance to an accessible green space was higher in the most deprived neighbourhoods (Q1 = 4.86 hm vs. Q5 = 5.46 hm; OR = 1.139, 95% CI: 1.084, 1.197).

Table 2 depicts the green space quality scores according to domain. All quality scores were negatively and significantly associated with neighbourhood deprivation, i.e., green space quality decreased as deprivation increases. For instance, the overall quality score (regardless of the computation method) exhibited a socioeconomic gradient, decreasing as neighbourhood deprivation increased (Q1 = 7.96, Q2 = 7.60, Q3 = 7.68, Q4 = 7.70, Q5 = 7.45; OR = 0.781, 95% CI: 0.729, 0.837).

Table 3 and Table 4 show the specific features available in the green spaces according to neighbourhood socioeconomic deprivation quintiles. Data from Table 3 indicated that the least deprived neighbourhoods had better access to green spaces designed for active recreation (Q1 = 97.1% vs. Q5 = 90.8%), with higher numbers of walking trails and courses for sports and playgrounds. Features related to green space environmental quality followed a less clear and sometimes opposed pattern (Table 3). For instance, gardens, water, and aesthetic features were more frequently found in the green spaces accessible to the more deprived neighbourhoods. Several detrimental features, such as the presence of vandalism, garbage, and graffiti, were still more common in the green spaces of the most deprived neighbourhoods.

Amenities such as play equipment (Q1 = 28.3% vs. Q5 = 24.0%), parking facilities (Q1 = 50.6% vs. Q5 = 28.7%), toilets (Q1 = 35.9% vs. Q5 = 30.0%), seating (Q1 = 96.8% vs. Q5 = 95.6%), clubrooms/meeting rooms (Q1 = 22.1% vs. Q5 = 14.4%), and drinking fountains (Q1 = 43.0% vs. Q5 = 39.2%), were more commonly present in the least deprived neighbourhoods. Finally, in what concerns safety issues, the domain with fewest items, we observed that least deprived neighbourhoods had green spaces with better visibility to the surrounding houses, and were more often surrounded by secondary roads.

The results from the multivariable ordinal regression are shown in Table 5. Although in the univariable models, shown in Table 1, most of the measures of geographic accessibility to green space were significantly associated with socioeconomic deprivation; in the multivariable model, only the mean distance to the green spaces within 800 m (OR = 1.156, 95% CI: 1.099, 1.215) remained significantly and positively associated with the deprivation quintiles (larger distances in more deprived neighbourhoods). On the other hand, apart from the activities-related quality score, all scores of green space quality—environmental quality (OR = 0.907, 95% CI: 0.853, 0.964), amenities (OR = 0.839, 95% CI: 0.791, 0.890), and safety (OR = 0.695, 95% CI: 0.618, 0.781)—showed a statistically significant and negative association with socioeconomic deprivation, indicating that green space quality is inferior in the more deprived neighbourhoods of the city. The fact that some measures were no longer associated with the deprivation quintiles in the multivariable model is likely due to the fact that most of the measures of green space geographic accessibility and quality were significantly correlated, as shown in correlation matrix provided in supplementary material (Table S3).

## 4. Discussion

Given the potential of green spaces to promote physical activity and health, the present study sought to evaluate the geographic accessibility and quality of the green spaces in the city of Porto (Northern Portugal) and investigated whether green space provision followed the principle of equality, or if, on the other hand, it favoured certain socioeconomic groups. Our findings indicated that there is differential access to green space in the city: geographical accessibility to green space was considerably better in the least deprived neighbourhoods, and most of the green space quality domains (safety, amenities, and environmental quality) were also superior in these neighbourhoods. On the other hand, green spaces in the more deprived neighbourhoods presented significantly more safety concerns, signs of damage, and lack of equipment to engage in active leisure activities (sports, walking), and had significantly less amenities such as seating, toilets, cafés, etc., that constitute important attractive elements for green space use.

Our results are consistent with findings reported elsewhere. Although the majority, roughly 80%, of the neighbourhoods in our study had an accessible green space, this share decreased as neighbourhood deprivation increased. Even though study designs are not entirely comparable, similar patterns were observed in Adelaide (Australia) [9], New Zealand [29], Shanghai (China) [66], Atlanta (USA) [25], as well as Indiana (USA), Los Angeles (USA) [35] and other U.S. cities [26,67]. Yet, a substantial amount of studies did not find differences in geographical accessibility but only in green space quality, which justifies the need to integrate both quality and proximity when addressing inequalities in green space provision. For instance, numerous studies found that, indeed, the availability of green space was higher in more deprived and minority communities, but these spaces presented more quality and safety concerns [33,34,35,36]. In Porto, socioeconomic differentials were present in these two dimensions of green space provision.

Although we cannot directly infer this from our study, our results suggest that that Porto residents of low socioeconomic position, who happen to be less physically active and more prone to chronic diseases [6,7,18], live in neighbourhoods with less green space. This confirms the “deprivation amplification hypothesis”, which states that places where people who have fewer personal resources reside often have fewer public resources that might buffer individual deprivation [20,26]. Several studies have linked lack of green space with physical inactivity, obesity [68], poor quality of life, and higher risk of death [69,70]. Taking this into account, our study showed that there are ample opportunities to increase physical activity, and thus to improve population health status, in low-income neighbourhoods. Also, an increasing body of research has shown that if fairly distributed, green spaces exert an “equigenetic” effect in several health outcomes [13,15,71], meaning that populations that are exposed to the greenest environments tend to have lower levels of health inequality. For instance, a Europe-wide study concluded that socioeconomic inequality in mental well-being was 40% narrower among respondents reporting good access to green areas, compared with those with poorer access, and no other community resource produced such levelling effect [71].

Interestingly, we found that, although the overall environmental quality was worst in the most deprived neighbourhoods of the city, certain items pertaining to the environmental quality domain—including the presence of water and aesthetic features and the existence of gardens—were more common in the most deprived communities. A similar pattern was also obtained by Vaughan et al. [36] in Kansas City, where medium-income neighbourhoods registered a higher number of aesthetic features. However, the origin of this “equitable difference” (i.e., better quality green space in areas usually considered disadvantaged) [22] is unclear. In Porto, this pattern could be related to two aspects: either targeted investments in more deprived communities or simply historical evolution of the green infrastructure. Regarding the first issue, the Porto city council has recently become much more aware of the impact of the built and natural urban environment on physical activity and population health [72,73], therefore the council might be adopting a strategy of providing better quality features in more disadvantaged areas; nonetheless, if true, this is only visible in certain features of the environmental quality domain. The explanation based on the historical evolution of the green space in the city is very plausible. A considerable amount of the most deprived neighbourhoods are located in the historical centre; green spaces in the historical centre date back to the 19th century and early 20th century, when these were viewed as meeting and convivial places for the upper social classes and the emerging bourgeois culture that demanded new lifestyles [51,74]. In that time, most green space investment went to aesthetic elements with significantly less to equipment for active recreation. Unfortunately, information on year of establishment of Porto green spaces is not complete, hampering our explorations of the interactions between time and green space quality. Indeed, it would be of extreme importance to understand the historical processes that shaped the sharp socioeconomic inequalities in green space provision we observed in Porto. Documental investigation (newspapers, ordinances, city plans), as conducted by Boone and colleagues in Baltimore [33], would help us to elucidate these pathways in future investigations.

Finally, we observed that, in the multivariable ordinal regression model, the quality score related with the availability of equipment for sports and exercise was not significantly related to socioeconomic deprivation when the other quality scores were taken into account. As shown in the correlation matrix from Table S3, the activities-related quality score was strongly and positively associated with the score related with presence of amenities (r = 0.432, *p* < 0.001), i.e., green spaces that provided spaces to engage in active leisure activities also happened to present various amenities (playgrounds, cafés, toilets).

The present study has a number of limitations that merit discussion. Firstly, the cross-sectional ecological design of this study prevents us from making any causal inference. It is not possible to answer the “which-came-first question”: neighbourhood deprivation or poor green space provision? We know that the installation of a green area generally comes with an increase in property and housing prices [75], and, therefore, might cause in-migration of the upper social classes and out-migration of the lower social classes. Our study was based on a single urban setting, which means (especially since the literature has shown large within-country variability [9,22] that our results cannot be straightforwardly extrapolated to other Portuguese and southern European cities, wherein green space provision policy and level of engagement in tackling socioeconomic differentials are possibly different. Due to data unavailability (Portuguese censuses do not include questions on race or ethnicity) we have not addressed ethnic/racial differentials in green space provision. Diverse studies have identified relevant inequalities in green space provision according to the ethnic and religious composition of the city tracts [22,25,76]. Yet, in our defence, immigration has a much smaller expression in Portugal (especially in Porto and Northern Portugal when compared with Lisbon and the South) than in other European and American cities [77], leading us to believe that addressing socioeconomic differentials is of greater importance. It is also important to refer that the chosen items of POST are debatable. Although it is documented that cafes [78], sport facilities [79] and/or gardens [78] are attractive features that promote green space use, it is possible that certain population subgroups (e.g., older adults, children/youth, minorities) do not feel the same. Yet, as we considered most POST items, it is unlikely those choices are driving our main results. Similarly, although dichotomization prior to index computation is a rather common procedure [80,81,82], this methodological approach is also arguable and might lead to some loss of information. Nevertheless, as most of POST items were originally dichotomous, we considered that this approach did not compromise our findings or conclusions. Finally, we did not examine whether access to green space was associated with actual physical activity, and whether these between-neighbourhood differences provided a potential explanation for the observed socioeconomic differentials in physical activity. Porto has three large population-based cohorts (http://ispup.up.pt/research/research-structures/) of adults, children and young adults, which means this question could be answered in future investigations.

Nevertheless, the present investigation does present several strengths. As far as we are aware, this is the first study addressing the presence of socioeconomic inequalities in green space access in Portugal, and one of the first in Southern Europe. To date, this topic has been overlooked in Europe, possibly because the environmental justice movement started and gained more expression in the USA and in Anglo-Saxon countries. We included a consistent objective definition of green space (green spaces that can be freely used by the population to engage in leisure and physical activities) and we considered the universe of public green areas and all neighbourhoods of the city, instead of relying on random samples of the universe. We addressed two important dimensions of green space access: geographic accessibility and quality, contrasting with a significant part of previous studies focused on the geographic accessibility dimension only. Finally, the quality of green space was evaluated with a well-established, validated tool with good psychometric properties that can be used in other contexts by academics and planners, allowing for comparisons through time and space.

## 5. Conclusions

In conclusion, this study revealed important socioeconomic inequalities in green space provision in a southern European setting. Porto residents from low socioeconomic positions seem to suffer from a double jeopardy: they lack both individual and community resources, which may limit their capacity to take control of their health and health-related behaviours, such as physical activity. Future research should extend to other cities in Portugal and Europe and should investigate whether other environmental correlates of physical activity (i.e., sports facilities, walkability, street safety, and maintenance) display the same pattern of socioeconomic inequality. 

Our findings are of great importance for those involved in urban planning and design and have the potential to assist in identifying target/priority areas and in designing environmental interventions to ensure adequate and equitable access to green space. Our study suggest that there is a need to improve accessibility and quality green space in Porto, especially in the more socioeconomically deprived areas of the city. As the urban environment is highly malleable and interventions and local authorities have some degree of autonomy of governance to respond to the needs of the population, improving and equalizing green space provision in Porto city seems to be an achievable target.

## Figures and Tables

**Table 1:** Geographic accessibility to green space according to neighbourhood socioeconomic deprivation quintiles.

Measures of Geographic Accessibility	All (*N* = 2064)	Q1 (*N* = 612)	Q2 (*N* = 137)	Q3 (*N* = 286)	Q4 (*N* = 284)	Q5 (*N* = 745)	Odds Ratio (95% CI) ^a^
Green spaces within 800 m (yes)	1656 (80.2)	551 (90.0)	111 (81.0)	210 (73.4)	219 (77.1)	565 (75.8)	0.550 * (0.451, 0.672)
No. of green spaces within 800 m (mean, SD)	2.07 (1.82)	2.22 (1.72)	2.75 (2.37)	1.92 (1.88)	2.02 (1.78)	1.90 (1.73)	0.924 * (0.885, 0.965)
Distance (in hm) to green spaces within 800 m (median, IQR)	5.14 (3.50)	4.86 (3.98)	4.37 (3.13)	5.53 (3.66)	5.44 (3.46)	5.46 (3.24)	1.139 * (1.084, 1.197)
Area of green space within 800 m per inhabitant (m^2^/inhab) (median, IQR)	2.31 (9.59)	3.82 (18.41)	3.28 (13.25)	1.75 (5.55)	1.99 (8.22)	1.74 (8.30)	0.999 (0.999, 1.000)

^a^ odds ratio and 95% confidence intervals obtained from the univariable ordinal regression; * *p* < 0.05; SD = standard deviation; Q1–Q5 = Quintiles 1–5; IQR = interquartile range.

**Table 2:** Green space quality scores according to neighbourhood socioeconomic deprivation quintiles.

Quality Scores	All (*N* = 2064)	Q1 (*N* = 612)	Q2 (*N* = 137)	Q3 (*N* = 286)	Q4 (*N* = 284)	Q5 (*N* = 745)	Odds Ratio (95% CI) ^a^
Activities	1.79 (0.93)	1.91 (0.89)	1.70 (0.80)	1.75 (0.88)	1.83 (0.93)	1.70 (0.99)	0.763 * (0.672, 0.868)
Environmental quality	8.88 (2.04)	8.93 (2.26)	8.60 (1.79)	8.87 (2.22)	9.11 (2.15)	8.83 (1.72)	0.934 * (0.889, 0.983)
Amenities	4.61 (2.20)	4.96 (2.29)	4.34 (1.71)	4.51 (2.12)	4.54 (2.35)	4.41 (2.15)	0.850 * (0.807, 0.898)
Safety	2.86 (0.95)	2.90 (1.05)	3.12 (0.71)	2.92 (0.79)	2.68 (1.02)	2.80 (0.92)	0.845 * (0.767, 0.932)
Total	18.14 (3.72)	18.70 (3.97)	17.77 (3.16)	18.05 (3.90)	18.17 (3.65)	17.74 (3.52)	0.902 * (0.875, 0.930)
Total domain weighted	7.69 (1.79)	7.96 (1.78)	7.60 (1.65)	7.68 (1.82)	7.70 (1.68)	7.45 (1.84)	0.781 * (0.729, 0.837)

^a^ odds ratio and 95% confidence intervals obtained from the univariable ordinal regression; * *p* < 0.05.

**Table 3:** Green space features according to neighbourhood socioeconomic deprivation quintiles.

Features	All (*N* = 2064)	Q1 (*N* = 612)	Q2 (*N* = 137)	Q3 (*N* = 286)	Q4 (*N* = 284)	Q5 (*N* = 745)	Correlation Coefficient ^a^
Proportion (%) of parks with a certain feature with relation to all the parks within 800 m from the neighbourhood.							
Domain: Activities							
Usage (active)	95.0	97.1	100.0	96.9	95.1	90.8	–0.114 *
No. of activities (≥2)	33.7	40.0	24.9	28.4	32.8	32.4	–0.049 *
Appropriateness to physical activities (high)	50.4	54.2	45.1	49.3	55.6	46.5	–0.045 *
Domain: Environmental quality							
Water features (yes)	44.9	36.7	37.4	41.5	51.4	53.3	0.132 *
No. of water features (≥2)	23.8	22.2	19.6	23.5	24.2	26.4	0.040 *
Aesthetic features (yes)	77.4	70.8	77.7	67.8	78.0	87.1	0.144 *
No. of aesthetic features (≥2)	42.1	32.5	34.5	37.1	52.3	51.2	0.152 *
Park size (large)	49.2	48.3	41.4	45.1	59.2	49.6	0.025
Tree density (high)	64.9	71.8	67.4	68.4	66.2	55.6	–0.114 *
Gardens (yes)	74.8	65.5	69.8	79.3	77.0	82.4	0.145 *
Paths (yes)	95.0	97.1	100.0	96.9	95.1	90.8	–0.114 *
Shade along paths (very good, good or medium)	68.1	71.2	61.0	72.4	75.4	62.3	–0.057 *
Watered grass (yes)	98.3	99.7	100.0	96.7	98.4	97.0	–0.077 *
Dog allowance (yes)	95.9	98.3	96.3	95.6	94.3	94.3	–0.077 *
Graffiti (no)	49.5	58.3	47.7	57.6	45.1	40.3	–0.134 *
Vandalism (no)	59.6	65.0	51.2	65.5	56.1	55.8	–0.065 *
Litter (no)	44.9	55.3	56.5	40.2	38.2	36.5	–0.150 *
Domain: Amenities							
Play equipment (yes)	25.0	28.3	19.9	22.9	25.1	24.0	–0.030 *
No. of play equipment (≥6)	13.4	19.5	12.7	16.0	12.4	7.1	–0.132 *
Picnic tables (yes)	19.2	19.8	8.5	16.7	17.1	23.3	0.041 *
Parking facilities (yes)	41.2	50.6	47.2	45.1	42.5	28.7	–0.164 *

^a^ Kendall’s tau-b; * *p* < 0.05.

**Table 4:** Green space features according to neighbourhood socioeconomic deprivation quintiles (continuation).

Features	All (*N* = 2064)	Q1 (*N* = 612)	Q2 (*N* = 137)	Q3 (*N* = 286)	Q4 (*N* = 284)	Q5 (*N* = 745)	Correlation Coefficient ^a^
Proportion (%) of parks with a certain feature with relation to all the parks within 800 m from the neighbourhood.							
Domain: Amenities							
Public access toilets (yes)	32.2	35.9	23.9	31.1	35.2	30.0	–0.035 *
Kiosk or café (yes)	14.4	14.6	9.5	13.6	14.8	15.5	0.016
Seating (yes)	96.5	96.8	98.4	97.3	96.3	95.6	–0.030 *
Clubrooms/meeting rooms (yes)	18.1	22.1	11.4	17.3	23.0	14.4	–0.059 *
Rubbish bins (yes)	92.4	94.9	95.5	90.0	89.7	91.2	–0.057 *
Dog litter bags (yes)	69.2	70.3	75.6	61.5	62.7	72.1	0.005
Drinking fountains (yes)	39.3	43.0	31.3	39.6	35.4	39.2	–0.025
Domain: Safety							
Lighting (yes)	93.6	94.5	96.8	92.7	89.0	93.9	–0.021
Visible roads (yes)	75.6	72.7	87.3	80.2	73.7	74.3	–0.001
Visible houses from centre (yes)	81.1	80.5	92.3	86.5	77.5	78.2	–0.038 *
Surrounded by secondary roads only (yes)	35.9	42.5	35.8	32.7	28.2	34.0	–0.069 *

^a^ Kendall’s tau-b; * *p* < 0.05.

**Table 5:** Association between green space geographic accessibility and quality and neighbourhood deprivation quintiles.

Variables	Odds Ratio (95% CI) ^a^ Multivariable Model
Geographic accessibility	
Distance (hm) to green spaces within 800 m	1.156 (1.099, 1.215) *
Quality scores	
Environmental quality	0.907 (0.853, 0.964) *
Amenities	0.839 (0.791, 0.890) *
Safety	0.695 (0.618, 0.781) *

^a^ odds ratio and 95% confidence intervals; * *p* < 0.05.

## Supplementary Materials

The following are available online at www.mdpi.com/1660-4601/14/8/916/s1, Table S1: Included and excluded items and recodification criteria. Table S2: Sensitivity analysis. Table S3: Correlation matrix: (A) Quality scores; (B) Geographic accessibility.
