# Supplementary material for: Socioeconomic Inequalities in Green Space Quality and Accessibility—Evidence from a Southern European City
Source: Int J Environ Res Public Health. 2017 Aug 15;14(8):916. doi: 10.3390/ijerph14080916 (PMC5580619; doi:10.3390/ijerph14080916)
Supplement: Supplementary file 1 [file ijerph-14-00916-s001.pdf]

Supplementary Materials: Socioeconomic Inequalities in Green Space Quality and Accessibility—Evidence from a Southern European City

Elaine Hoffimann, Henrique Barros and Ana Isabel Ribeiro *

Table S1. Included and excluded items and recodification criteria.

Item	Status	Recodification
	General information	
1.Reference	Excluded	-
2. Address	Excluded	-
3.Area	Included	Dichotomized according to the median value<br>1 = larger<br>0 = smaller
4.Postcode	Excluded	-
5.Geocode (X,Y)	Excluded	-
6. Year of establishment of POS:	Excluded (24% missing info)	-
	Domain: Activities	
7. Type of usage	Included	1 = active formal/informal use<br>0 = passive use only
8. For what type of activities is the space designed?	Included	Sum of the number of offered activities and then dichotomized according to the median value:<br>1 = ≥2<br>0 = ≤1
49. To what extent do you agree or disagree with each of the following statements regarding this POS?<br>POS is interesting for walking<br>POS is suitable for casual ball sports<br>POS is suitable for cycling	Included	Level of appropriateness to physical activities<br>Sum of the Likert scale and then dichotomized according to the median value:<br>0 = low<br>1 = high
	Domain: Environmental Quality	
9. Is the POS on the beach/river foreshore?	Excluded<br>(could artificially overestimate socioeconomic inequalities)	-
10. Are there water features within the POS?	Included	Not required
11.Type of water feature	Included	Sum of the number of water features and then dichotomized according to the median value:<br>1 = ≥2<br>0 = 1
12. Estimate the percentage of the POS occupied by the water feature(s)?	Excluded<br>(all green spaces had up to 25%)	-
13a. Are there other aesthetic features in the POS?	Included	Not required

13b. Which of the following features are present?	Included	Sum of the number of aesthetic features and then dichotomized according to the median value:<br><br>1 = ≥2<br>0 = 1
14. Are there trees in this POS?	Excluded<br>(all green spaces had trees)	-
15. Estimate the approximate number of trees present	Included	A measure of tree density was computed according to a classification matrix based on the green space size (terciles of area) and number of trees, as follows:<br>0 = large size areas with 1-100 trees plus medium size areas with 1-50 trees.<br>1 = large size areas with more than 100 trees plus medium size areas with more than 50 trees plus small size areas
16. Where are the trees placed?	Excluded (unpredictable impact in green space use)	-
17. Are there gardens in this POS?	Included	Not required
18a. Are there walking paths or cycleways within or around the POS?	Included	1 = paths (walking or dual use)<br>0 = no paths
18b. Shade along paths	Included	1 = very good, good or medium<br>0= no paths / poor or very poor
19. Describe the placement of paths within the POS	Excluded (unpredictable impact in green space use)	-
20. Is there evidence that the grass is watered?	Included	Not required
21. Are dogs allowed?	Included	1 = Yes (at all times, at certain times or not specified)<br>0= No
22. Is access for dogs:	Excluded (unpredictable impact in green space use)	-
23. Is graffiti present?	Included	1 = No<br>0 = Yes
24. Is vandalism evident?	Included	1 = No<br>0 = Yes
25. Is there litter throughout the POS?	Included	1 = No<br>0 = Yes
Domain: Amenities		
26. Is children's play equipment present?	Included	Not required
27. What items of play equipment are present?	Included	Sum of the number of play equipment features and then dichotomized according to the median value:<br>1 = ≥6<br>0 = ≤5
28. What is the playground surface?	Excluded (unpredictable impact in green space use)	-

29. Is playground shaded?	Excluded (all were shaded)	-
30. Are barbecues present?	Excluded (absent in all green spaces)	-
31. Are picnic tables present?	Included	Not required
32a. Are there parking facilities serving the POS?	Included	Not required
32b. Estimate the number of bays	Excluded (proportional to the park size)	-
33. Are there public access toilets?	Included	Not required
34. Is there a kiosk/café present?	Included	1 = 7 days per week/weekdays only/weekends only<br>0 = No
35. Is there access to public transport within one block of POS?	Excluded (present in all green spaces)	-
36. Is there seating present?	Included	Not required
37. Are there clubrooms/meeting rooms present?	Included	Not required
38. Are rubbish bins present?	Included	Not required
39. Are dog litter bags provided?	Included	Not required
40. In how many locations in POS are dog litter bags present?	Excluded (proportional to the park size)	-
41. Are there taps or other water sources accessible for dogs?	Excluded (absent in all green spaces)	-
42. Are drinking fountains present?	Included	Not required
	Domain: Safety	
43. Is there lighting within the POS? (i.e., not just street lighting)	Included	Not required
44. Where is the lighting located?	Excluded (unpredictable impact in green space use)	-
45. From the centre of the POS, how visible are surrounding roads?	Included	1 = clearly or partially visible<br>0 = not visible
46a. From the centre of the POS, how visible are the surrounding houses?	Included	1 = clearly or partially visible<br>0 = not visible
46b. How many of these houses overlook the park?	Excluded	-
46c. Is there any area of the POS where you are unable to clearly see surrounding houses?	Excluded	-
47. Are all roads surrounding the POS minor roads or cul-de-sacs?	Included	Not required
48a. Does the major road/s have a zebra crossing to assist access to the POS?	Excluded (present in all green spaces)	-
48b. Does the major road/s have a pedestrian crossing with signals to assist with access to the POS?	Excluded (present in all green spaces)	-

Table S2. Sensitivity analysis (400 m distance threshold).

(A) Measures of green space geographic accessibility and quality according to neighbourhood deprivation quintiles (descriptive statistics).

Variables	All (*N* = 2064)	Q1 (*N* = 612)	Q2 (*N* = 137)	Q3 (*N* = 286)	Q4 (*N* = 284)	Q5 (*N* = 745)
Geographic accessibility						
Green spaces within 400 m (yes)	1012 (49.0)	347 (56.7)	74 (54.0)	147 (51.4)	133 (46.8)	311 (41.7)
No. of green spaces within 400 m (mean, SD)	0.69 (0.90)	0.83 (0.98)	1.10 (1.37)	0.61 (0.66)	0.62 (0.78)	0.56 (0.79)
Distance (in hm) to greenspaces within 400 m (median, IQR)	2.25 (1.79)	2.01 (1.72)	2.31 (1.90)	1.87 (1.97)	2.31 (1.66)	2.47 (1.46)
Area of greenspace within 400 m per inhabitant (m^2/inhab) (median, IQR)	0.00 (1.76)	0.25 (2.84)	0.20 (2.85)	0.00 (1.78)	0.00 (1.44)	0.00 (0.80)
Green space quality scores						
Activities (mean, SD)	1.86 (0.95)	1.95 (0.87)	1.60 (0.77)	1.87 (0.94)	2.02 (0.92)	1.76 (1.08)
Environmental quality (mean, SD)	8.86 (2.11)	8.69 (2.16)	8.34 (1.49)	9.42 (2.94)	9.45 (2.22)	8.77 (1.62)
Amenities (mean, SD)	4.76 (2.24)	4.99 (2.20)	4.21 (1.60)	4.82 (2.12)	5.09 (2.74)	4.53 (2.24)
Safety (mean, SD)	2.88 (0.95)	3.06 (0.98)	3.23 (0.56)	2.94 (0.75)	2.41 (1.17)	2.69 (0.89)
Total (mean, SD)	18.36 (3.79)	18.69 (3.69)	17.38 (2.88)	19.06 (4.73)	18.97 (3.81)	17.75 (3.58)
Total domain weighted (mean, SD)	7.81 (1.77)	8.10 (1.67)	7.46 (1.62)	8.18 (1.88)	7.90 (1.58)	7.39 (1.88)

SD = standard deviation; IQR = interquartile range.

(B) Association between green space geographic accessibility and quality and neighbourhood deprivation quintiles (univariable and multivariable ordinal regression).

**Variables**	**Odds ratio (95%CI)** [a] **Univariable**	**Odds ratio (95%CI) Multivariable**
Geographic accessibility		
Green spaces within 400 m (yes)	0.634 (0.542, 0.742) *	
No. of green spaces within 400 m	0.758 (0.694, 0.829) *	0.860 (0.743, 0.996) *
Distance (hm) to accessible green spaces	1.269 (1.145, 1.405) *	1.288 (1.161, 1.429) *
Area of green space within 400 m per inhabitant (m^2/inhab)	0.996 (0.994, 0.999) *	
Quality scores		
Activities	0.875 (0.771, 0.994) *	
Environmental quality	0.975 (0.925, 1.027)	0.939 (0.884, 0.996) *
Amenities	0.929 (0.881, 0.978) *	0.857 (0.807, 0.910) *
Safety	0.714 (0.634, 0.804) *	0.586 (0.507, 0.678) *

[a] odds ratio and 95% confidence intervals; * $p < 0.05$.

Table S3. Correlation matrix (Kendall's tau-b correlation coefficient) of the green space characteristics: (A) Quality scores; (B) Geographic accessibility.

**Quality Scores**	**Activities Score**	**Environmental Quality Score**	**Amenities Score**	**Safety Score**	**Geographic Accessibility**	**Green Spaces within 800m (yes)**	**No. of Green Spaces within 800m**	**Distance (m) to Green Spaces within 800m**	**Area of Green Space within 800m per Inhabitant (m²/inhab)**
Activities score	1.000	0.168 $p < 0.001$	0.432 $p < 0.001$	–0.189 $p < 0.001$	Green spaces within 800m (yes)	1.000	0.624 $p < 0.001$	–	0.602 $p < 0.001$
Environmental. quality score	0.168 $p < 0.001$	1.000	0.235 $p < 0.001$	–0.370 $p < 0.001$	No. of green spaces within 800m	0.624 $p < 0.001$	1.000	0.053 $p = 0.003$	0.539 $p < 0.001$
Amenities score	0.432 $p < 0.001$	0.235 $p < 0.001$	1.000	–0.193 $p < 0.001$	Distance (m) to green spaces within 800m	-	0.053 $p = 0.003$	1.000	–0.027 $p = 0.127$
Safety score	–0.189 $p < 0.001$	–0.370 $p < 0.001$	–0.193 $p < 0.001$	1.000	Area of green space within 800m per inhabitant (m²/inhab)	0.602 $p < 0.001$	0.539 $p < 0.001$	–0.027 $p = 0.127$	1.000

© 2017 by the authors; licensee MDPI, Basel, Switzerland. This article is an open access article distributed under the terms and conditions of the Creative Commons by Attribution (CC-BY) license (http://creativecommons.org/licenses/by/4.0/).
